# World Antimalarial Resistance Network (WARN) IV: Clinical pharmacology

**DOI:** 10.1186/1475-2875-6-122

**Published:** 2007-09-06

**Authors:** Karen I Barnes, Niklas Lindegardh, Olumide Ogundahunsi, Piero Olliaro, Christopher V Plowe, Milijaona Randrianarivelojosia, Grace O Gbotosho, William M Watkins, Carol H Sibley, Nicholas J White

**Affiliations:** 1 Division of Clinical Pharmacology, Department of Medicine, University of Cape Town, Cape Town, South Africa; 2 Mahidol Oxford Research Unit, Faculty of Tropical Medicine, Mahidol University. Bangkok Thailand; 3 Nuffield Department of Medicine, University of Oxford, Oxford, UK; 4 UNICEF/UNDP/WB/WHO Special Programme for Research & Training in Tropical Diseases, World Health Organisation, Geneva, Switzerland; 5 School of Medicine, University of Maryland, Baltimore, USA; 6 Institut Pasteur de Madagascar, Antananarivo City, Madagascar; 7 Department of Pharmacology and Therapeutics, College of Medicine, University of Ibadan, Ibadan, Nigeria; 8 Department of Genome Sciences, University of Washington, Seattle, WA, USA

## Abstract

A World Antimalarial Resistance Network (WARN) database has the potential to improve the treatment of malaria, through informing current drug selection and use and providing a prompt warning of when treatment policies need changing. This manuscript outlines the contribution and structure of the clinical pharmacology component of this database. The determinants of treatment response are multi-factorial, but clearly providing adequate blood concentrations is pivotal to curing malaria. The ability of available antimalarial pharmacokinetic data to inform optimal dosing is constrained by the small number of patients studied, with even fewer (if any) studies conducted in the most vulnerable populations. There are even less data relating blood concentration data to the therapeutic response (pharmacodynamics). By pooling all available pharmacokinetic data, while paying careful attention to the analytical methodologies used, the limitations of small (and thus underpowered) individual studies may be overcome and factors that contribute to inter-individual variability in pharmacokinetic parameters defined. Key variables for pharmacokinetic studies are defined in terms of patient (or study subject) characteristics, the formulation and route of administration of the antimalarial studied, the sampling and assay methodology, and the approach taken to data analysis. Better defining these information needs and criteria of acceptability of pharmacokinetic-pharmacodynamic (PK-PD) studies should contribute to improving the quantity, relevance and quality of these studies. A better understanding of the pharmacokinetic properties of antimalarials and a more clear definition of what constitutes "therapeutic drug levels" would allow more precise use of the term "antimalarial resistance", as it would indicate when treatment failure is not caused by intrinsic parasite resistance but is instead the result of inadequate drug levels. The clinical pharmacology component of the WARN database can play a pivotal role in monitoring accurately for true antimalarial drug resistance and promptly correcting sub-optimal dosage regimens to prevent these contributing to the emergence and spread of antimalarial resistance.

## Background

The overall objective of the Worldwide Antimalarial Resistance Network (WARN) database is to improve the treatment of malaria, by informing drug selection and use and providing a prompt warning of when treatment policies need changing [1]. This database should include data on the clinical pharmacology of antimalarial drugs in addition to their therapeutic efficacy, and the molecular and in vitro markers of resistance. The clinical pharmacology component of the database is essential to ensure optimal dosing with currently available and newly introduced antimalarial drugs through defining the correlation between drug concentration and clinical and parasitological response. This requires three key questions to be answered: firstly, what is the profile of "therapeutic" drug concentrations that needs to be sustained to eradicate the plasmodial biomass? Secondly, are these drug concentrations over time being achieved in the majority of all target groups, including infants, pregnant women, and those with prevalent co-morbid diseases (especially HIV/AIDS, malnutrition) or are there important sub-groups being under (or over) dosed? For example inadequate drug levels have been repeatedly found in pregnant women and young children [2-8]. Despite the antimalarial drug concentrations often being approximately half of those considered therapeutic, there has not yet been any dosage regimen adjustment made to ensure that these vulnerable populations are adequately dosed. Third, if current dosing is found to be sub-optimal, can the regimen be adjusted safely to ensure therapeutic dosing?

The determinants of therapeutic response are multi-factorial. The ability of available antimalarial pharmacokinetic data to inform optimal dosing is constrained by the small number of patients studied, with even fewer (if any) studies conducted in the most vulnerable populations. The variability in biological matrix (plasma, venous whole blood, venous capillary blood), analytical methodology used and the marked inter-individual pharmacokinetic variation of some antimalarials (e.g. artemisinins) create further challenges, in particular the need for effective quality control of laboratory assays of drug concentrations in biological samples, particularly blood – the site of action of antimalarials. In the past, some attempts at standardization were made without much success. Now that a global database on the clinical pharmacology of antimalarials is being planned, it is the appropriate time to consolidate effort in this field. Individual pharmacokinetic studies are almost invariably underpowered in characterising the factors that influence antimalarial pharmacokinetic parameters. By pooling all available pharmacokinetic data, while paying careful attention to the methodology used, the limitation of small (and thus underpowered) individual studies may be overcome and the factors that contribute to inter-individual variability in pharmacokinetic parameters defined. Similarly if there were important drug-concentration related toxicities, which could well have been missed due to the paucity of the available data, these could be best recognized through this database.

A better understanding of the pharmacokinetic properties of antimalarials and a more clear definition of therapeutic drug levels would allow more appropriate use of the term "antimalarial resistance", as it would indicate when treatment failure is not caused by parasite resistance but is the result of inadequate drug levels, through sub-optimal dosing, poor adherence, poor quality of the antimalarial, poor absorption or poor metabolism to the active metabolite (for example with amodiaquine [9,10]). The clinical pharmacology component of the WARN plays a pivotal role in accurately monitoring for true antimalarial drug resistance and promptly correcting sub-optimal dosage regimens to prevent these contributing to the spread of antimalarial resistance.

## Objectives

The main objective of the clinical pharmacology component of this database is to ensure rational use and optimal dosing regimens in all target groups for currently available and newly introduced antimalarial drugs. Achieving this objective requires an understanding of the pharmacokinetic profile of each important component antimalarial (including active metabolites) in each target group, including infants, pregnant women, those with co-morbid disease (especially HIV/AIDS, tuberculosis and malnutrition). However, merely describing pharmacokinetic parameters in each target population would not be enough to optimize dosage regimens, as this requires a clear definition of "therapeutic drug levels", i.e. the pharmacokinetic parameters required to achieve an adequate clinical and parasitological response (ACPR), specifically in non-immune patients. The efficient analysis of the global database will facilitate prompt recognition of:

1. target populations in whom a substantial proportion fail to achieve therapeutic drug concentrations

2. target populations in which pharmacokinetic data on key antimalarials are lacking or are inadequate

3. when or where the recommended dose results in a substantial number of treatment failures, despite achieving drug concentrations that were previously defined as "therapeutic", and whether this problem can be safely addressed by increasing the dose, or whether a change in policy is required.

4. Geographic variations which might have genetic or environmental origins.

In addition to the potential of the database to support optimization of dosage regimens, it creates the opportunity to simplify regimens based on age or weight/height, and even to explore the potential of using pharmacokinetic measures to assess adherence. Importantly, by better defining these information needs and criteria of acceptability of pharmacokinetic-pharmacodynamic studies, the database should contribute to improving the quantity, relevance and quality of subsequent studies.

## Challenges

### Access to unpublished data

There are considerable obstacles that need to be overcome to achieve the ambitious objectives of the clinical pharmacology component of the WARN database. These include the paucity of published pharmacokinetic studies. These have generally been small detailed studies conducted in too few patients to inform rational optimal dosing regimens. Some pharmacokinetic data (particularly the studies conducted during drug development) are not in the public domain. These data are held by the manufacturer, both commercial pharmaceutical companies and increasingly by non-governmental organizations and Public-Private Partnerships, such as the Medicines for Malaria Venture (MMV) and the Drugs for Neglected Diseases Initiative (DNDi). It is in the interest of public health that such data, and other unpublished pharmacokinetic data, is rapidly included in the WARN Clinical Pharmacology Database where it may be freely accessed. This requires that obstacles to such groups contributing their data to the database are identified, and true partnership between stakeholders established to resolve how these obstacles can be overcome. Given the limited PK data currently available, it is considered advantageous that all available pharmacokinetic data, despite their limitations, be included in the WARN Clinical Pharmacology Database and that the subsequent analysis be used to determine which results are consistent, both in terms of pharmacokinetic parameters and factors that influence these.

### Neglected populations

Most pharmacokinetic studies are conducted in healthy volunteers and adult patients, with the most vulnerable populations (infants, pregnant women, those with HIV/AIDS co-morbidity, malnourished patients) systematically excluded from these studies. Researchers and funders need to be encouraged to address these gaps urgently, given the substantial impact that under-dosing of these vulnerable groups would have on malaria morbidity and mortality and on the selection and spread of antimalarial resistance. Similarly, standard dosage regimens may be associated with toxic drug levels being achieved in other vulnerable populations.

### Quality assurance and quality control

**Figure 1:**
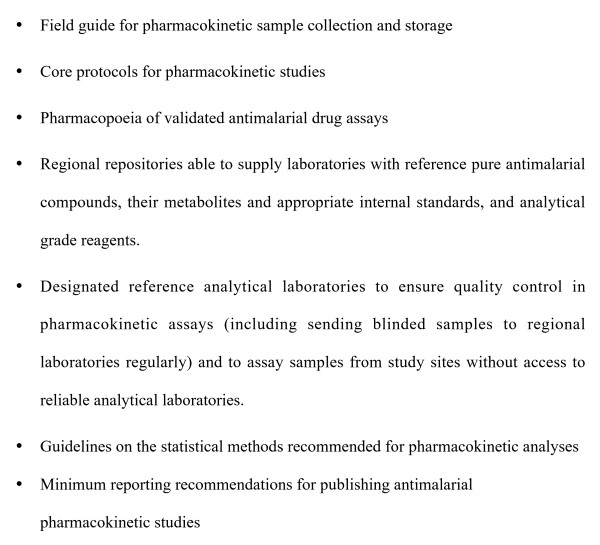
Parameters for modeling calibration curves.

The main limitation in pooling individual patient data is the variability in assay and analysis methodology between pharmacokinetic studies. Another limitation is if different biological matrices are used for different studies. There are not always straightforward concentration relationships between different matrices. This complicates the comparison of results from different laboratories and studies, and may invalidate pooling of data and meta-analysis of results. It may be impossible to know whether the pharmacokinetic properties in study populations actually differ or whether apparent differences merely reflect variability in the accuracy of the methods used. This can be addressed through method standardization or preferably, for more stable antimalarials, by the establishment of a quality assurance process where blinded samples are sent to all participating laboratories and the results of their assays compared with the drug concentrations measured by the reference laboratories. This would be essential even for laboratories that have ISO accreditation or Good Laboratory Practice certification. Similar Quality Control schemes operate in other disciplines such as haematology, biochemistry, and immunology. Global participation in this scheme would be facilitated by availability of a field guide (Standard Operating Procedures) for sample collection and storage, core protocols for pharmacokinetic (pharmacodynamic studies), a pharmacopoeia of validated antimalarial drug assays (including regression models), and regional repositories able to supply laboratories with reference pure antimalarial compounds, their metabolites and appropriate internal standards, and analytical grade reagents (Appendix 1). It is important to standardize not only the actual assay methodology but also the standard/calibration curve relationship (i.e. regression model) for quantification of unknown samples (see Figure 1). Similarly, guidelines are needed on the statistical methods recommended for the analysis of pharmacokinetic data and capacity building of statisticians in malaria endemic countries, particularly for the complex and time consuming population pharmacokinetic analyses. Future comparisons of pharmacokinetic studies would be facilitated by defining the minimum requirements for publishing antimalarial pharmacokinetic studies.

The assay methodology is a key determinant of the accuracy of the results. In most cases Mass Spectrometric and High Performance Liquid Chromatographic assays are more likely to give reliable results than the Bioassay or simple chemical or chromatographic methods, although early Mass Spectrometric assays may have been compromised by ion suppression [11]. Validity can be further assessed by considering the reported recovery, coefficients of variation (for quality control samples), back-calculated concentrations for calibration standards, regression model, stability, lower limits of quantification, sample handling, volume of sample, biological matrix, anticoagulant, duplicate assay, inter- and intra-assay variability, and data handling. These factors could be used to rate the quality of the pharmacokinetic data on a scale of 0 (poor) to 3 (excellent), rather than including each individual variable in the database. This could be done through the development of an algorithm for rating validity for each assay. Alternatively, a statistical approach to pooled data can be taken to determine whether the laboratory identity is an independent predictor of pharmacokinetic parameters, once other known PK determinants are adjusted for.

### Structure and content of the WARN Clinical Pharmacology Database

The WARN Clinical Pharmacology Database will have two components: one summarizing all available pharmacokinetic (pharmacodynamic) data and the second containing individual patient data on the pharmacokinetics (and where available pharmacodynamics) of antimalarial drugs. The available summary pharmacokinetic data will be collated into an open access database as rapidly as possible, to inform the finalising of the structure of the individual-patient database and identify gaps in the data (e.g. vulnerable target populations for whom no pharmacokinetic data are currently available).

The most valuable, longer term contribution of the Clinical Pharmacology component of a global antimalarial drug resistance database is likely to be individual patient data, as this should overcome the problem of small underpowered individual pharmacokinetic studies and ultimately is essential for making progress toward defining *in vivo *therapeutic ranges and modelling resistance, both of which are needed to optimize useful therapeutic life of the few available antimalarial drugs. Linking individual patient pharmacokinetic data with the demographics of those studied will allow us to define the determinants of these pharmacokinetic parameters.

The database needs to include study patient characteristics, methodology for determining the pharmacokinetics of each antimalarial, and where available, antimalarial treatment response (pharmacodynamics). The primary and secondary variables in the database are summarized in Tables 1 and 2.

**Table 1:** Primary parameters to be included in WARN Pharmacokinetic Database

**Patient characteristics**	**Antimalarial**	**Methodology**	**Pharmacokinetic parameters**
Malaria status [1]	Generic name	Sampling times (to include sampling on day of recurrence of parasitemia)	Day 7 concentration (median, IQR, range)
Age	Dose (total)	Sample Matrix (e.g. Venous blood, capillary blood, filter paper spot, breast milk, placenta, urine, saliva)	AUC (median, IQR, range)
Weight	Manufacturer	Assay method	Pharmacokinetic model
Gender	Route of administration	Assay Validity [3]	
Sample size		Sample volume	
Special Risk Group [2]		Sample storage (temperature and duration)	

Footnotes to Table 1

**Malaria status**: Healthy volunteer; Asymptomatic Pf malaria; Intermittent preventive treatment (IPT); Uncomplicated Pf malaria; Moderately severe malaria; Uncomplicated hyperparasitaemia; Severe malaria; Uncomplicated P. vivax malaria; Prophylaxis; Other non-malaria disease.

2. **Special Risk Group**: Pregnancy (trimester); Infants; Pre-school; Co-morbid disease (HIV/AIDS; Tuberculosis; malnourished; obese).

3. **Assay Validity**: Retrospective data: Validity rating; Prospective data: pre-defined QC requirements

**Table 2:** Secondary parameters to be included in WARN Pharmacokinetic Database, if available.

**Patient characteristics**	**Antimalarial**	**Methodology**	**Pharmacokinetic parameters**
Height	Directly Observed Therapy (DOT) [1]	Method for separating free drug	Primary parameters: Absorption rate constant (Ka); Elimination Rate constant (Ke), Volume of distribution; Clearance rate.
Vomiting	Batch number & Expiry Date	Sample handling	Secondary parameters: Cmax, tmax, elimination half-life;
Diarrhoea	Method of Administration [2]		Total vs free drug
Baseline parasite density	Co-administration [3]		Metabolites
Haematocrit, Bilirubin, Creatinine, Albumin (alpha1 glycoprotein, lipoprotein)			
Ethnicity			

Footnotes to Table 2

**DOT**: All doses supervised; First dose supervised; Unsupervised.

2. **Administration**: Whole tablets; Divided tablets; Crushed tablets.

3. **Co-administration**: Water; Fat content; Sugar water; Concomitant medication

## Patient characteristics

A thorough understanding of what factors influence antimalarial drug exposure would be facilitated by the inclusion of all pharmacokinetic studies in the database. The effect of disease would be assessed by comparison of pharmacokinetics in normal healthy volunteers with malaria patients. The effect of disease severity would be explored by comparing parasitaemic patients with asymptomatic, uncomplicated, moderately severe and severe malaria – with careful definition of these classifications. The pharmacokinetics of antimalarials administered as intermittent preventive therapy to pregnant women, infants and children, needs to be determined specifically as they may differ substantially both from those in normal healthy volunteers and in non-pregnant adult patients with malaria. As vast quantities of antimalarials, particularly chloroquine, are administered to patients infected with non-falciparum malaria parasites, particularly *Plasmodium vivax*, pharmacokinetic studies in these populations are essential for optimizing antimalarial drug use.

Given marked variability in the quality of antimalarials available [12], it is essential to specify the details regarding the antimalarial drug used in each pharmacokinetic study including manufacturer, source, expiry date (and ideally batch number and reference method used to ensure tablet content). It would be desirable also to include testing of the actual content of antimalarial drug in several tablets (or ampoules etc.) with standardized methodology. Similarly the exact method of drug administration needs to be documented, noting which of the doses were supervised, how tablets were administered to young children (as these are often crushed in sugar solution, jam or banana which may alter bioavailability) and, if relevant (e.g. lumefantrine), the quantity of fat administered together with the antimalarial dose. The route and time(s) of administration and actual dose administered, including when doses are repeated following vomiting, need to be specified. Patients in pharmacokinetic studies who require repeated dosing (usually following vomiting) should continue to have drug concentrations measured throughout the follow up period, provided the time and dose of repeated administration is recorded accurately. Current policies to exclude these patients from further studies preclude evidence-based guidelines for the management of these patients, even though vomiting is commonly reported among children with malaria. The weight of the patient is required to specify the mg per kg dose administered, and can identify those patients that are likely to be malnourished or obese; this assessment can be made more accurate by inclusion of height in the database as this would allow for the calculation of Body Mass Indices (BMIs).

Age and weight influence pharmacokinetics, so characterization of these effects is necessary for the development of simplified yet effective dosage regimens by age category. In areas of moderate and high intensity malaria transmission, age is an important surrogate indicator of the probability of acquired partial immunity, which needs to be included when assessing the pharmacokinetic-pharmacodynamic relationship. Similarly, pregnancy status (and trimester) needs to be included as immunity is reduced during pregnancy, and physiological changes during pregnancy are likely to alter the pharmacokinetics of most antimalarials. A recent history of vomiting or diarrhoea prior to oral treatment may be associated with lower bioavailability [13]. Recording of co-morbid diseases, particularly HIV/AIDS and malnutrition, would identify whether any alteration in treatment dosage or policy is indicated for patients with these conditions.

Pharmacokinetic studies with commonly used concomitant medication (particularly antiretroviral treatments, anti-emetics, antihistamines) will identify clinically significant drug interactions, while administration of, for example, antipyretics or antibiotics with antimalarial activity needs to be documented for reliable interpretation of the therapeutic response. Variations in antimalarial pharmacokinetics by region or ethnic group might point to potential pharmacogenetic differences in drug metabolism indicating when pharmacogenomic studies would be useful. Useful supporting laboratory data include measures of haematocrit, bilirubin, creatinine and albumin. Other proteins such as the main binding protein for basic drugs alpha acid glycoprotein are relevant for quinine and chloroquine, while artemisinins also bind to lipoproteins [14-16].

## Measuring drug concentrations over time

The selection of sample matrix (e.g. venous blood, capillary blood, plasma, breast milk, placenta, urine) will depend on the drug and the study context and objectives. Capillary blood of fixed volume collected into calibrated capillary tubes and dried on to filter paper is the most feasible sample for use in field studies, and such assays have been validated for chloroquine, quinine, halofantrine, piperaquine, amodiaquine, sulphadoxine/pyrimethamine, atovaquone, mefloquine and proguanil [7,17-23]. The accuracy of these assays depends on the filter paper used (and its pre-treatment, when needed) and relies on careful training to ensure, for example, an accurate volume of sample, avoiding diluting the sample with interstitial tissue fluids by excessive squeezing of the patient's finger, and preventing contamination by touching the filter paper after touching the study drug. A simple field guide providing simple SOPs is needed to optimize sample collection and storage. Where protein binding is substantial, it is ideal to measure both total and free drug, although this is not possible for whole blood spots dried onto filter paper. Furthermore, the concentration of free drug for drugs with substantial protein binding (i.e. >99%) is very low and will thus in many cases be limited by assay sensitivity. There are currently no validated assays for capillary blood spots on filter paper for pyronaridine, lumefantrine or the artemisinin derivatives.

The selection of sampling times depends on the pharmacokinetic properties, particularly the elimination half-life, of each antimalarial drug, the context of the study and the therapeutic objective of treatment. Although time to reach therapeutic concentrations is of critical importance in treating severe malaria, for the treatment of uncomplicated malaria, an adequate clinical and parasitological response requires the persistence of levels that exceed the minimum inhibitory concentration (a concentration resulting in a parasite multiplication rate of <1/cycle) for at least four 48-hour asexual blood cycles in non-immune patients. The day 7 drug concentration has been shown to be the most important single concentration, in terms of correlation with the area under the concentration time curve and or association with treatment response, for lumefantrine, piperaquine, quinine, mefloquine and sulphadoxine-pyrimethamine [7,17,24-27]. As day 7 is a routine visit in therapeutic efficacy studies [28], measuring drug concentrations at least at this time point is recommended for all medium- to long acting drugs (i.e. drugs with half-lives greater than 12 hrs). This requires drug assays that are sensitive enough to quantify antimalarial drug levels accurately at least until this time point. Treatments with shorter elimination half-lives require more frequent earlier sampling. Further pharmacokinetic studies are needed to define optimal sampling times for the treatment of *P. vivax *(and other non-falciparum species).

The respective roles of conventional and population pharmacokinetic approaches need to be considered when selecting timing of sample collection. Intensive sampling is needed initially for each antimalarial and in each important target population. Subsequently, population pharmacokinetics can be used to minimize the number of times blood needs to be collected from an individual patient, thereby allowing more patients to be included in the study. Population pharmacokinetic analysis is complex and time consuming, taking between six and 12 months per drug. Extensive capacity building (particularly training in statistical modelling) is needed for population pharmacokinetic modelling to be more widely used, particularly in malaria endemic countries. Occasionally, the population pharmacokinetic approach will not succeed in modelling the pharmacokinetics of a particular antimalarial. Standardization in reporting will also be needed. Given the skew distribution of most pharmacokinetic parameters, these should preferably be reported as medians, inter-quartile ranges and ranges. Although summary measures are most often reported, the extremes of the range are more important in exploring the pharmacokinetic determinants of treatment failure and toxicity. This creates need for accurate characterization of the variability in of pharmacokinetic parameters.

## Pharmacokinetic-pharmacodynamic relationships

Defining *in vivo *therapeutic levels requires analysing associations between drug levels and the risk of recurrent parasitaemia, recrudescence and re-infection [29]. For those antimalarials where the parasite mutations associated with resistance have been defined, this analysis should be stratified by genotype [30]. It is useful to determine the drug level at the time of failure, even when this was not a routine pharmacokinetic sampling time, although clinical signs and symptoms at this time need to be recorded as illness itself may affect pharmacokinetic parameters, such as the volume of distribution. Comparison of the pharmacokinetic parameters at the time of late clinical and late parasitological failure can be used to measure this effect. Population pharmacokinetics may be helpful to predict what the antimalarial drug level was likely to have been when parasitaemia became patent.

The association between drug exposure and the rate of reduction in parasite density (Parasite reduction ratios (PRRs), Parasite Clearance Times (PCTs), Time to clearance of 50% and 90% of parasites (PC50, PC90) are expected to be the earliest measures for detecting sub-optimal exposure to artemisinins and decreasing artemisinin efficacy as these drugs uniquely affect ring stage clearance. Monitoring the association between drug levels and post-treatment fever is useful both as a determinant of some pharmacokinetic parameters and as a secondary efficacy endpoint; however, the use of antipyretics complicate the interpretation of this association.

To define the upper limit of the therapeutic range, all adverse events need to be recorded and related to drug exposure. Particular attention should be paid to gastro-intestinal disturbances following oral treatment as this could reduce drug bioavailability. Recording of time to vomiting and the effect of this on drug levels, including when dosing is repeated following vomiting, is essential to inform how patients who vomit after oral treatment should be managed.

Lastly, a better understanding of how the pharmacokinetic profiles of various artemisinin-based combinations influence gametocyte carriage and the selection of resistant re-infections, would help to predict the therapeutic life of an antimalarial. This could help inform the selection of treatment policies, and help drug developers prioritize development of new antimalarials that have the best chance of remaining effective for longest.

## Conclusion

The public health benefits of recent dramatic increases in spending on antimalarials create a particular urgency for ensuring that patients with malaria receive the best treatment in the correct dose. The investment in a global clinical pharmacology database would create a valuable resource that would make a substantial contribution to improving the treatment of malaria, which is currently compromised by increasing antimalarial resistance and the lack of pharmacokinetic data or sub-optimal dosing in vulnerable populations, particularly pregnant women and young children. The success of the database depends on willingness to share all available pharmacokinetic data and prompt, intelligent and unbiased analysis and interpretation of the determinants of pharmacokinetic parameters and the association of these with therapeutic response.

## Authors' contributions

KB drafted the manuscript based on prior discussions with WW and NJW and NL, OO, PO, CP, MR, SG, WW, and NJW revised the manuscript critically for important intellectual content. All authors have given final approval of this manuscript.

## Appendix 1: Tools to facilitate participation in Clinical Pharmacology Database

○ Field guide for pharmacokinetic sample collection and storage.

○ Core protocols for pharmacokinetic studies.

○ Pharmacopoeia of validated antimalarial drug assays.

○ Regional repositories able to supply laboratories with reference pure antimalarial compounds, their metabolites and appropriate internal standards, and analytical grade reagents.

○ Designated reference analytical laboratories to ensure quality control in pharmacokinetic assays (including sending blinded samples to regional laboratories regularly) and to assay samples from study sites without access to reliable analytical laboratories.

○ Guidelines on the statistical methods recommended for pharmacokinetic analyses.

○ Minimum reporting recommendations for publishing antimalarial pharmacokinetic studies
